# The ligamentous cervical instability etiology of human disease from the forward head-facedown lifestyle: emphasis on obstruction of fluid flow into and out of the brain

**DOI:** 10.3389/fneur.2024.1430390

**Published:** 2024-11-27

**Authors:** R. A. Hauser, D. Matias, B. Rawlings

**Affiliations:** Caring Medical Florida, Fort Myers, FL, United States

**Keywords:** cervical instability, cervical ligament injury, cervical lordosis, dysstructure, jugular vein compression, intracranial hypertension

## Abstract

Ligamentous cervical instability, especially ligamentous upper cervical instability, can be the missing structural cause and/or co-morbidity for many chronic disabling brain and systemic body symptoms and diagnoses. Due to the forward head-facedown lifestyle from excessive computer and cell phone usage, the posterior ligament complex of the cervical spine undergoes a slow stretch termed “creep” which can, over time, lead to cervical instability and a breakdown of the cervical curve. As this degenerative process continues, the cervical curve straightens and ultimately becomes kyphotic, a process called cervical dysstructure; simultaneously, the atlas (C1) moves forward, both of which can lead to encroachment of the structures in the carotid sheath, especially the internal jugular veins and vagus nerves. This obstruction of fluid flow can account for many brain diseases, and compression and stretch of the vagus nerve for body diseases, including dysautonomia. This article describes the consequences of impaired fluid flow into and out of the brain, especially venous flow through the internal jugular veins, leading to intracranial hypertension (formerly called pseudotumor cerebri). Cervical structural, internal jugular vein, and optic nerve sheath measurements are presented from a retrospective chart review of 227 consecutive patients with no obvious cause for 1 of 8 specific brain or mental health symptoms—anxiety, brain fog, concentration difficulty, depression/hopelessness, headaches, obsessive thoughts, panic attacks, and rumination on traumatic events. A case example is given to demonstrate how cervical structural treatments can open up internal jugular veins and improve a patient’s chronic symptoms.

## Introduction

Many chronic brain disorders are thought to occur primarily because of brain physiology, including brain fog, fatigue, cognitive decline, emotional lability, and mental health conditions such as depression, generalized anxiety disorder, bipolar disorder, personality disorders, depersonalization, and dissociative disorders. There is a mental health crisis in the United States that continues to worsen. The percentage of American adults that suffer from a mental illness has increased from 17.7% (40.7 million people) in 2008 to 20% (57.8 million people) in 2021 (1, 2). According to the World Health Organization, it expects depression, which is the most common mental disorder, to become the largest single healthcare burden by 2030 (3). The meteoric rise of cognitive and mental health disorders may be explained by many factors, but one potential cause could be a breakdown of the cervical neck curve and ligamentous cervical instability due to the forward head-facedown lifestyle (FH-FDL) from poor posture while using electronic devices, leading to impairment of fluid flow out of the brain via compression of the internal jugular veins in the carotid sheath.

*The body craves a balance between stability and mobility*. When the body is exposed to trauma or chronic low-grade abnormal forces, it leads to ligamentous cervical instability (LCI) and ultimately broken neck structure, termed “cervical dysstructure”: 2 common conditions that are often overlooked or missed by current static supine diagnostic testing methods. Its incidence is increasing with the current ever-prevalent FH-FDL of looking at computers and down at cell phones and tablets. This practice is especially damaging for children, who have a heavier head-to-neck strength ratio than adults. While looking at electronic devices can appear innocuous, it is not so. As the posterior ligamentous complex of the neck stretches, LCI can develop, which is a progressive condition that can lead to cervical dysstructure. Simply put, LCI can lead to a broken neck structure.

LCI may have humble beginnings with prolonged stretching from FH-FDL posture, and the first sign may be neck tightness. If left unchecked, it can progress into neurocatastrophic consequences as the cervical supporting structures weaken and the carotid sheath and/or spinal cord, and the fluids contained within them, become compressed, torqued, and stretched. The 2 most common mechanisms by which severe symptoms arise are venolymphatic drainage compression—leading to intracranial hypertension/increased brain pressure (IBP, formerly referred to in medical literature as pseudotumor cerebri) and alterations of brain function—and cervicovagopathy (cervical-induced vagus nerve injury or signal interference), causing dysautonomia and systemic inflammation, both of which lead to dysfunctional body homeostasis. The most common spinal instability involves the spine’s most mobile segment, the atlantoaxial joint (C1-C2), whose pathophysiology may unlock the cause and solution to neurodegenerative conditions, including dementia.

Mechanical stability of the spine depends on its ability to maintain alignment and provide protection to the neural, vascular, and other structures it encloses during physiological loading so that there is no harm to any of these tissues and no symptoms are produced (4, 5). *Clinical ligamentous cervical instability is an inability of the cervical ligaments to maintain individual, adjacent, or global vertebral alignment when subjected to increased forces by* var*ious postures, positions, and/or motions that alter bony, soft tissue, and/or neurovascular alignment and function such that symptoms result.* LCI causes the bones to move in such a way that destructive forces are placed on adjacent tissues and joints, but also on the myriad vital structures that run through the neck. It is to be distinguished from hypermobility, which entails increased motion of bony and/or soft tissue structures without tissue injury or symptoms.

The potential seriousness of LCI is amplified when one considers that all of the major neurovascular structures from the body that enter and leave the brain and brainstem, including the autonomic ganglia, do so through the neck. The neck is thus a conduit for fluid and nerve flow that run the body through the brain and brainstem. LCI can disrupt this fluid and nerve flow and can explain many resulting chronic symptoms, disorders, syndromes, and diagnoses that plague humanity.

## Cervical osseous-ligamentous anatomy

The cervical spine consists of 7 vertebrae and may be considered as 2 distinct regions: upper cervical (C0-C2), also known as the craniocervical junction (CCJ), and lower cervical (C3-C7). The lower cervical spine is connected by intervertebral discs and is thus inherently more stable than the CCJ. The C0-C1 joint is relatively stable, whereas the most mobile section of the spinal column is the C1-C2 joint because of the peg-like structure—the dens of C2—around which the arch of C1 rotates, giving it enormous flexibility. The C1-C2 joint sits between 2 more stable areas (C0-C1 and C2-C7), contributing to its being the most commonly unstable joint in the cervical spine. This precariousness is compounded by the fact that the average head weighs 10–12 pounds and sits on the 2-ounce C1 (atlas).

It is important to remember that ligaments are the fasteners of the bones, the connective tissues that hold adjacent bones together. *Most ligaments of the body are capsular ligaments, meaning they are the primary structures of the joint capsule that stabilizes the joint*. Ligaments can weaken over time if too much force is put upon them. The degeneration of ligaments (ligamentosis) is common in osteoarthritis (6, 7).

**Figure 1:**
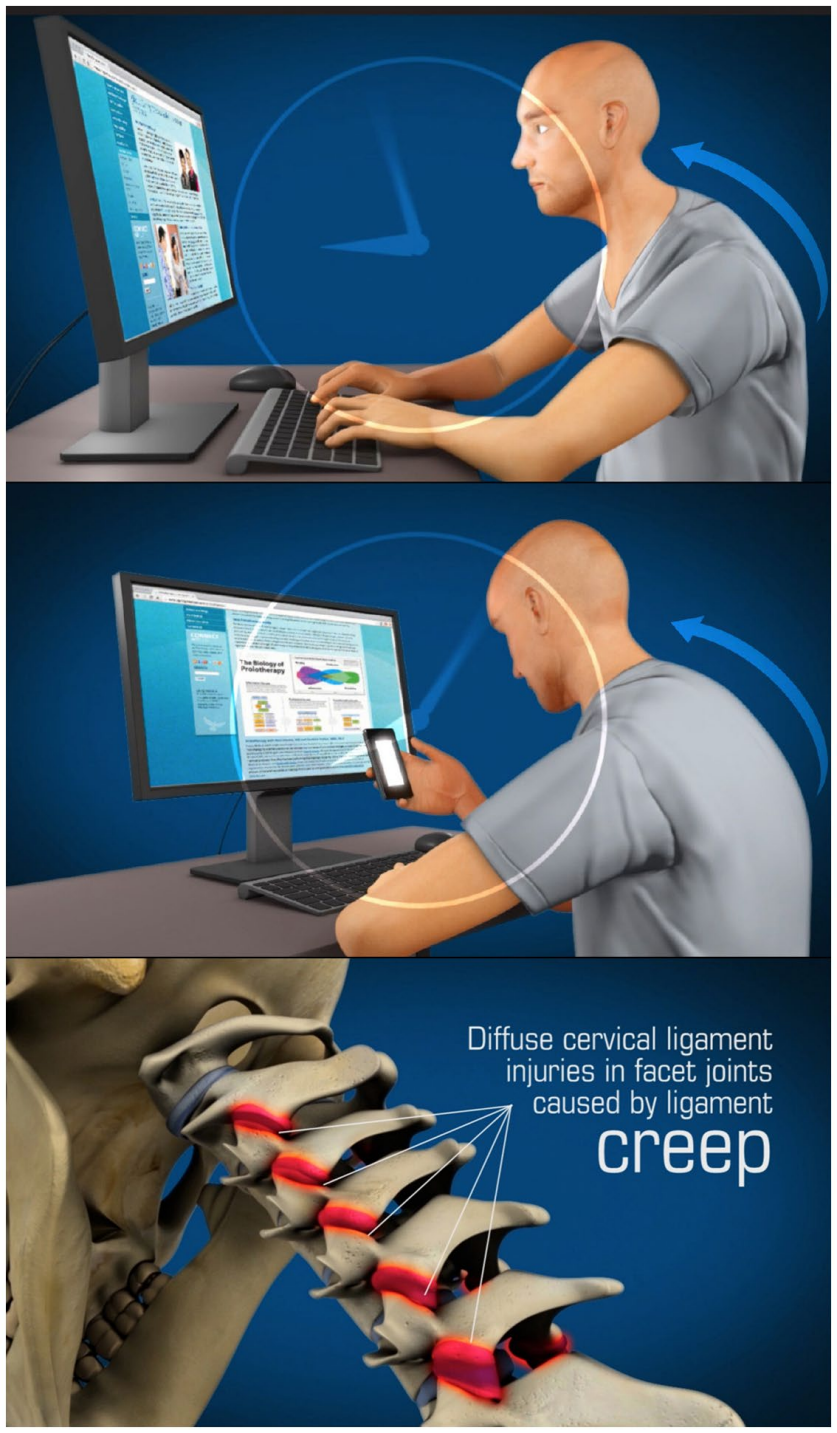
Forward head posture from hours of computer work and texting, resulting in cervical ligament laxity. “Creep,” which is a term signifying the slow stretching of ligaments, most commonly occurs by a forward head posture from computer work or looking at a smartphone.

The major cervical ligaments that are overstretched by the FH-FDL in the process known as “creep” are those of the posterior ligament complex (PLC), especially the capsular ligaments of the facet (zygapophyseal) joints, the major joints in the cervical spine. All ligaments posterior to the vertebral body, including the capsular ligaments, ligamentum flavum, posterior longitudinal, interspinous, intertransverse, and supraspinous ligaments, are considered part of the PLC and are stretched during flexion, the very motion in FH-FDL (see Figure 1). Conversely, any ligaments located anterior to the vertebral body, including the anterior longitudinal ligament, are stretched during extension. In regard to the 4 motions of the cervical spine—flexion, extension, axial rotation, and lateral flexion—it is only the capsular ligaments of the facet (apophyseal) joints that inhibit all 4 motions (8, 9). Since the facet joint is the fulcrum of all motions of the cervical spine (except rotation, in which it is the atlanto-dens joint), when the cervical capsular ligaments of the facet joints weaken, it is potentially possible that all neck motions are increased, which becomes evident when *motion* diagnostic scans are performed.

The neck, primarily the upper cervical spine, must support the head and keep it balanced and safe at all times, for bodily and brain functions. The CCJ (occiput to C2) is without intervertebral discs and relies primarily on ligamentous stabilizers for support. The atlantoaxial is the most mobile joint in the vertebral column. It is also the most vulnerable to FH-FDL, as well as injury during whiplash and the quick head turning that occurs during head trauma when the skull hits another object (10).

The fulcrum of head rotation is the atlantoaxial joint (C1-C2) (11). The primary ligamentous supports of the lateral facet joints of C1-C2 are the capsular ligaments, whereas the atlanto-dens joint is held together by the transverse ligament and alar ligaments. The C1-C2 atlantoaxial joint provides 50% of cervical spine rotational mobility as the arch of C1 rotates around the dens of C2 (12). When stable, C1-C2 allows some flexion, but almost no lateral flexion. Lateral flexion in this joint is one of the hallmarks of facet atlantoaxial instability. In the medical literature, atlantoaxial instability generally refers to the connection between the atlas and the dens of axis, which can be described as dens atlantoaxial instability. This is an important distinction because dens atlantoaxial instability is more of a surgical lesion, whereas facet atlantoaxial instability can often be treated by conservative measures such as chiropractic care, physiotherapy, and Prolotherapy (13, 14).

Protection against anterior translation of the atlas comes from the capsular ligaments posteriorly and the transverse ligament anteriorly. Capsular ligament injury of C1-C2 results in facet atlantoaxial instability, whereas injury of the transverse and alar ligaments causes dens atlantoaxial instability. Injury to the atlantoaxial capsular ligaments causes a dramatic increase in lateral bending and axial rotation motion (43 and 159%, respectively), whereas transverse ligament disruption significantly increases the anterior atlanto-dens interval (15).

**Figure 2:**
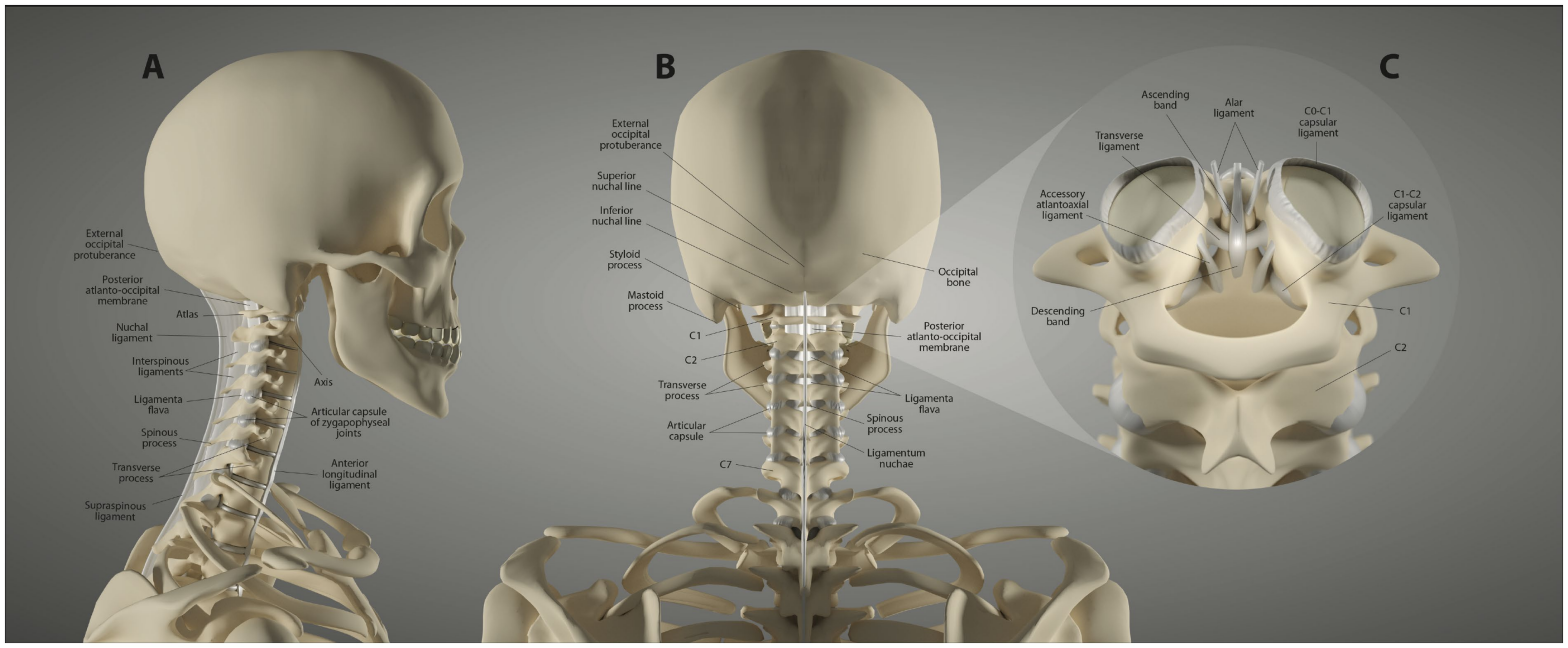
Ligament anatomy of the neck. **(A)** Lateral view. **(B)** Posterior view. **(C)** Upper cervical posterior view.

The remaining cervical vertebrae are the more typical vertebrae of the spine, each of which consists of a body, transverse processes and pedicles, and adjoining intervertebral discs. The discs make this part of the cervical spine inherently more stable. As with the CCJ, the fulcrum of motion is at the facet, with the C4-C5 facet joints being the fulcrum for neck flexion (16). The superior and inferior articular processes articulate bilaterally to form the facet joints at each level, which are true synovial joints. The lower cervical segments are chiefly supported by the intervertebral discs, anterior longitudinal ligament, posterior longitudinal ligament, ligamentum flavum, and the facet capsular ligaments (see Figure 2).

## The innate importance of the cervical lordotic curve

Cervical lordosis is the anterior convexity of the cervical spine from the foramen magnum to the thoracic spine and is caused by its wedge-shaped vertebrae. Cervical lordosis maximizes the spine’s ability to handle forces, maintains global spinal alignment, and supports and balances the head. The ideal sagittal alignment of the cervical spine is lordotic as measured in the sagittal plane by the C2-C7 Cobb angle of 20–35° (17–20). The cervical lordosis depth is normally between 7 and 17 mm as measured by the Borden method (21, 22). This keeps the center of mass of the cranium posterior of the lordotic curve, reducing the forces on the structures in the neck.

The neurovascular and soft tissue support in the neck is best in a relaxed (innate) configuration with minimum tension, which optimizes function. If there is a disruption of cervical lordosis, the axial load (cranial center of motion) shifts anteriorly and will continue to do so until cervical kyphosis results (23). This kyphosis can lead to stretching and lengthening of the spinal cord and other nerves that travel in the neck, including the vagus nerve, affecting nerve impulse conduction (24). This shift in axial load will cause cervical posterior ligamentous creep, further stretching the facet capsular ligaments, explaining why LCI is a progressive disorder.

While there are many causes of cervical ligament injuries, including whiplash and head traumas, by far the most common today is due to the FH-FDL from too much time in flexion (25, 26). This constant head and neck flexion places enormous forces on the neck-supporting structures. A neck flexed at 40° while looking at a cell phone triples the forces at C2 (27). In one study, researchers found that the forces on the cervical nervous tissue increased from 2 pounds per square inch in the neutral posture to 40 pounds per square inch in flexion (28). An adult head weights 10 to 12 pounds in the neutral position. As the head tilts forward, the forces seen by the neck surge progressively up to 60 pounds at 60° (29). With the neck flexed, the maximum load the neck can handle drops drastically (25–50%) (30, 31). Eventually, the neck structure becomes broken, and the person loses their normal lordotic curve.

## Cervical dysstructure degenerative cascade

**Figure 3:**
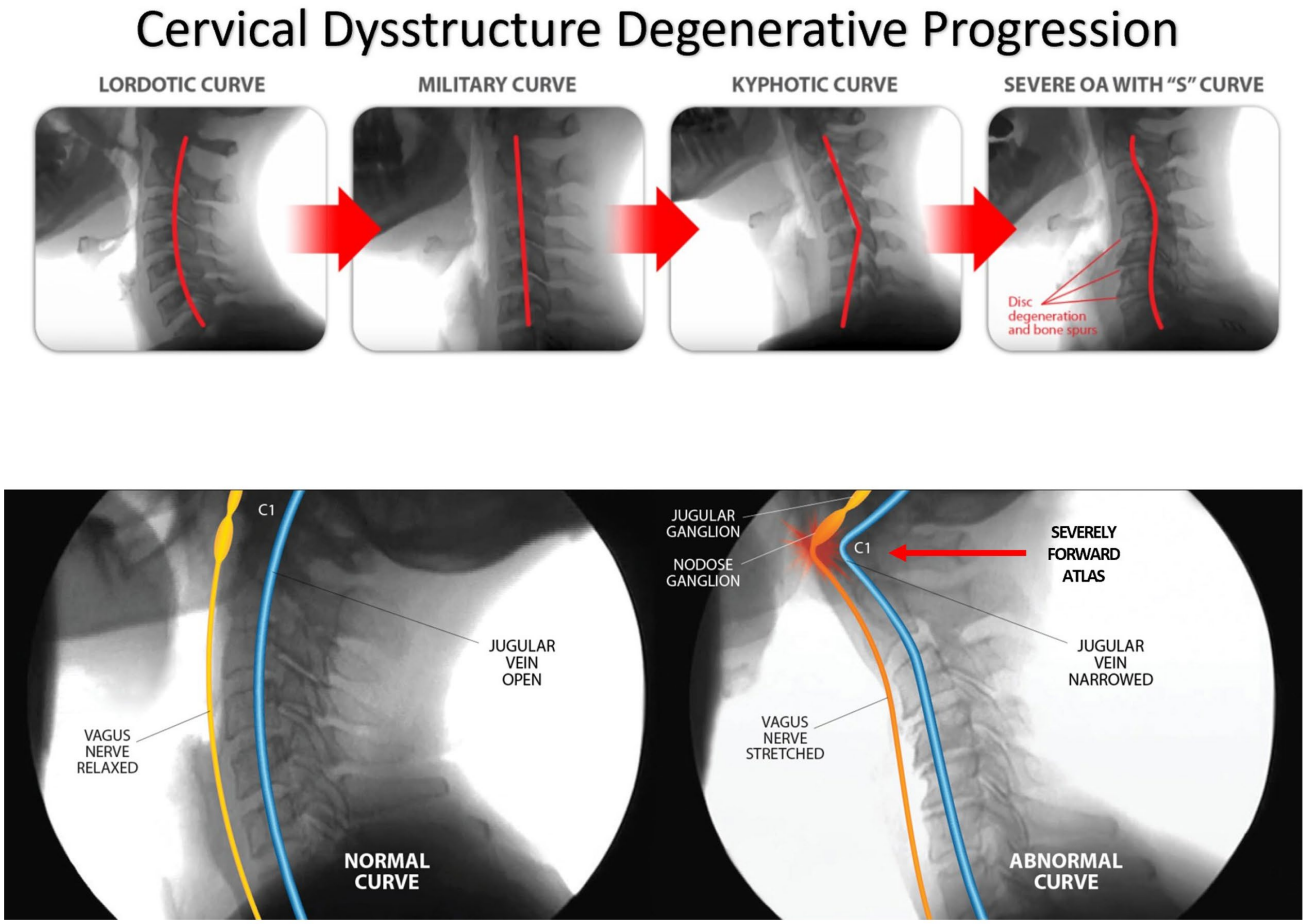
Ligamentous cervical instability is a progressive disorder causing a normal lordotic curve to break down, ultimately leading to an “S” or “sigmoid” curve and potentially compressing the structures in the carotid sheath, including the vagus nerves and internal jugular veins.

**Figure 4:**
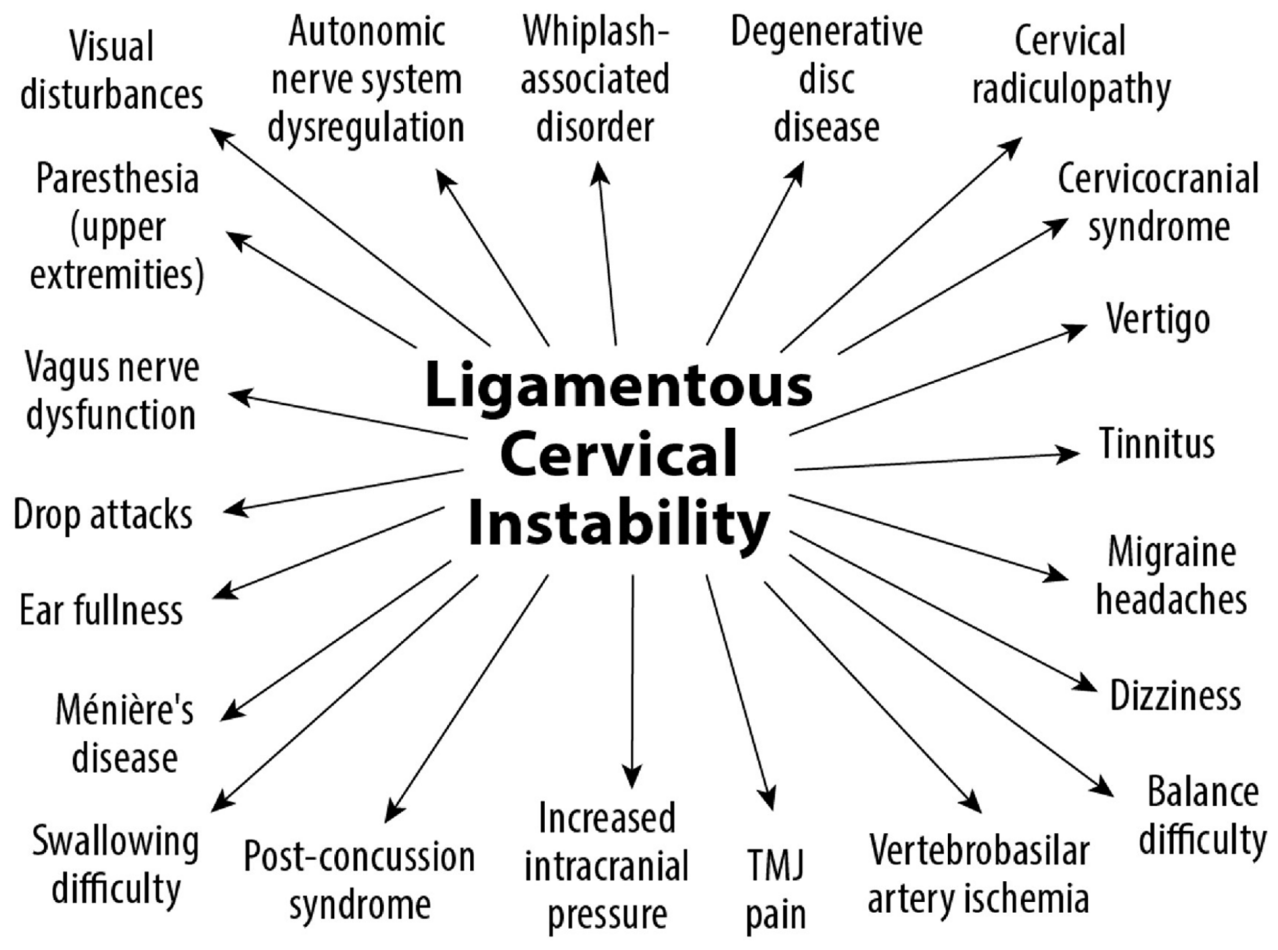
The many potential syndromes and symptoms caused by ligamentous cervical instability.

LCI is a progressive disorder leading to a breakdown of the cervical lordotic curve, a condition we call cervical dysstructure (see Figure 3). Cervical instability causes many symptoms, including cracking, clicking, and grinding in the neck with movement, as well as neck stiffness, muscular tension and weakness (feeling of the head being too heavy), and headache. What is not as well appreciated is that it can potentially cause a host of other disabling symptoms, including vertigo, dizziness, tinnitus, migraine headaches, and brain fog (32). LCI causes human disease by 2 primary mechanisms: it interrupts nerve signals (i.e., spinal cord and vagus nerves) and obstructs fluid flow to and from the brain and body. Following Occam’s razor, we know that the simplest solution is normally the correct one, so for a complicated patient with myriad unexplained symptoms, ligamentous cervical joint instability could be the best explanation (33) (see Figure 4).

In the cervical spine, *the body will always choose stability over motion* to protect the adjacent neurovascular structures. This is why long-term osteophytes form, and eventually, osteoarthritis causes a limiting of motion. When cervical ligament injury is present, the body has 3 main mechanisms to try limiting motion to protect the neurovascular structures: muscle tension or spasms, joint swelling, and osteophyte formation (34). Through the ligament-muscular reflex, muscle spasms protect neurovascular structures when ligament injury is present. When the facet capsular ligaments undergo a noxious stretch, research suggests a strain threshold as low as 10% stretch for activation. The nerve afferents that innervate it are also stretched, triggering neuronal signaling to the central nervous system and many of the 30+ stabilizing cervical muscles, as muscle insertions have been found to cover nearly 23% of the capsule area in the cervical spine (35–37). A hallmark of ligamentous upper cervical instability (LUCI) is tension or tenderness in the suboccipital and posterior neck muscles, as they are activated by the ligament-muscular reflex (38, 39). The neuronal signaling from the ligaments and tight muscles can also cause referral pain to the head and arm.

The degenerative cascade will proceed if the forces continue to exceed the cervical spine’s ability to handle them. As the FH-FDL progresses, so do the LCI and cervical dysstructure. Eventually, the flexion of the lower cervical spinal segments (C2–C7) causes compensatory hyperextension of the suboccipital (C0–C2) to maintain horizontal gaze (40, 41). It is also possible for bony hypertrophy to occur, leading to spinal stenosis and possible myelopathic or radicular symptoms. Other possible consequences of the continual forces and destabilization of the cervical spine are torsion, stretch, and compression of the vital neurovascular structures of the central and autonomic nervous system nerves/ganglia and blood vessels on their way to or from the brain.

**Figure 5:**
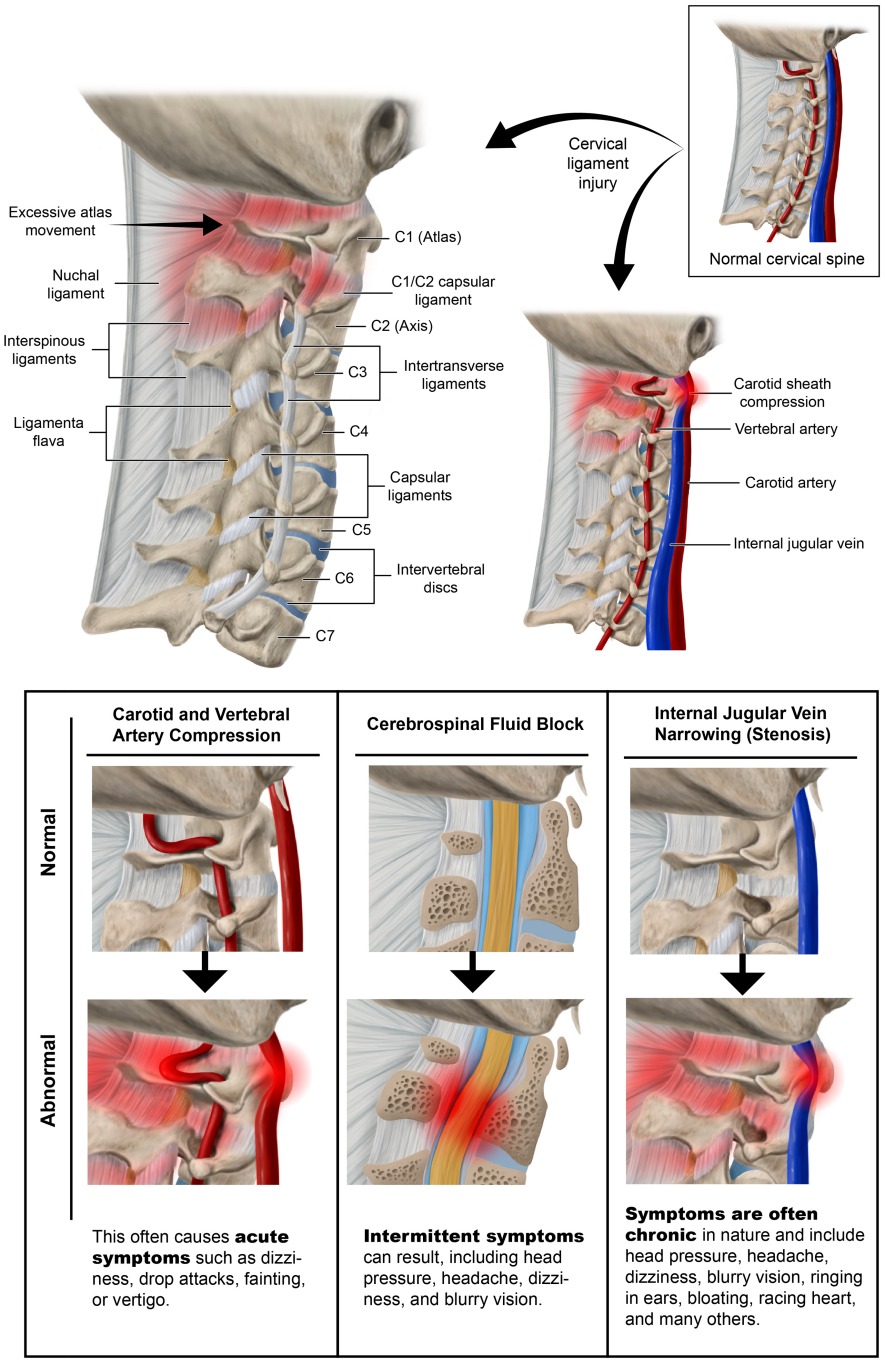
Ligamentous upper cervical instability can affect all 3 fluid flows through the neck.

The Gestalt of cervical spine destabilization and resultant kyphotic curve and tissue destruction is degenerative disc disease and cervical spondylosis, causing traction on the cervical spinal cord and brainstem, potential stretch and compression of the vagus nerves, and restrictions of the fluid flow into and out of the brain from the carotid and vertebral arteries, internal jugular vein (IJV), and cerebrospinal fluid (CSF) (42–44) (see Figure 5). The detrimental consequences of cervical dysstructure can be many, and include increased forces on the PLC, fixation or fusions of vertebral motion segments, increased forces on vertebral segments that still move, dysfunctional vertebral motions, significant muscle tightness and shortening, altered mechanical tension on neurovascular structures, and narrowing of vital spaces all throughout the neck.

## Cervical structural analysis: documentation of ligamentous cervical instability by upright, positional, and motion scanning

Humans spend the larger part of each day in the upright position, the very position that causes the majority of symptoms, yet most diagnostic tests are performed with the patient in the supine, resting position, the very position that provides relief. Scanning in the upright position can show the brain, brainstem, and cervical spine under the effects of gravity, and its alterations in blood flow, venous drainage, and CSF flow can also be seen while the person is upright. Many times, symptomology occurs with a specific head/neck position, such as flexion. Thus, while scanning the person (preferably during motion) when they are upright *or in their symptomatic position, upright x-rays, MRI, or positional computerized tomography (CT) scans* may be preferred, as static or supine scan abnormalities often do not correlate with symptoms (45–47). Dynamic MRI in the CCJ was found to be able to detect cases of cord compression that were not seen by static supine MRI (48). In a study involving 1,200 patients, cerebellar tonsillar descent (Chiari) of at least 1.0 mm was 4 times more likely to be diagnosed by an upright MRI vs. one supine (49). Cerebellar tonsillar ectopia was found 2.5 times more often in whiplash patients when an upright MRI was done vs. one that was supine. In this study and others, soft tissue lesions are found in the upper cervical region about 3–5 times more often when an upright (especially with flexion/extension views) vs. recumbent scan is performed (50–53).

**Figure 6:**
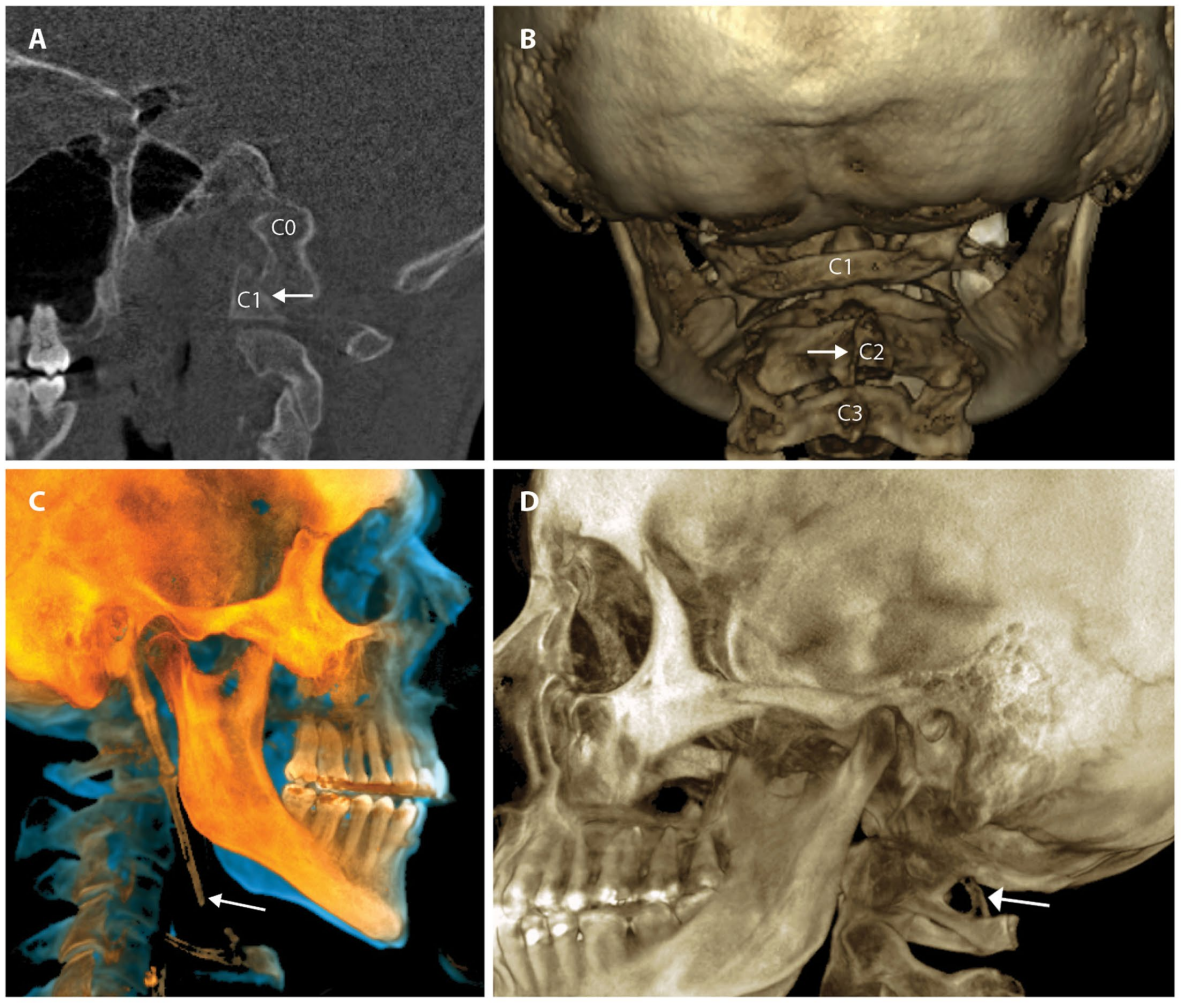
Common pathology seen by upright cone beam CT scan of head and neck. **(A)** CO-C1 instability. **(B)** Cervical spine misalignments. **(C)** Elongated styloid. **(D)** Ponticulus posticus.

Cervical structural analysis in the upright position and/or with motion allows the clinician to see the motion of vertebral bones in real time and discover previously hidden pathology. The 2 modalities that can be utilized for this imaging are digital motion x-ray (DMX) and upright cone beam CT scanning. Digital motion x-ray (videofluoroscopic) examination of the cervical spine has been shown to provide a high degree of diagnostic accuracy for the identification of vertebral instability in patients with chronic pain stemming from whiplash trauma (54). CT scanning of the cervical spine with 3-D reconstruction in various positions, including flexion, extension, and/or rotation, is another way to document cervical instability and misalignments that are missed by more traditional means (55). Cone beam CT scanning can document elongated styloid bones, which can compress the carotid sheath, and thus the IJVs, with head rotation and flexion (see Figure 6).

**Figure 7:**
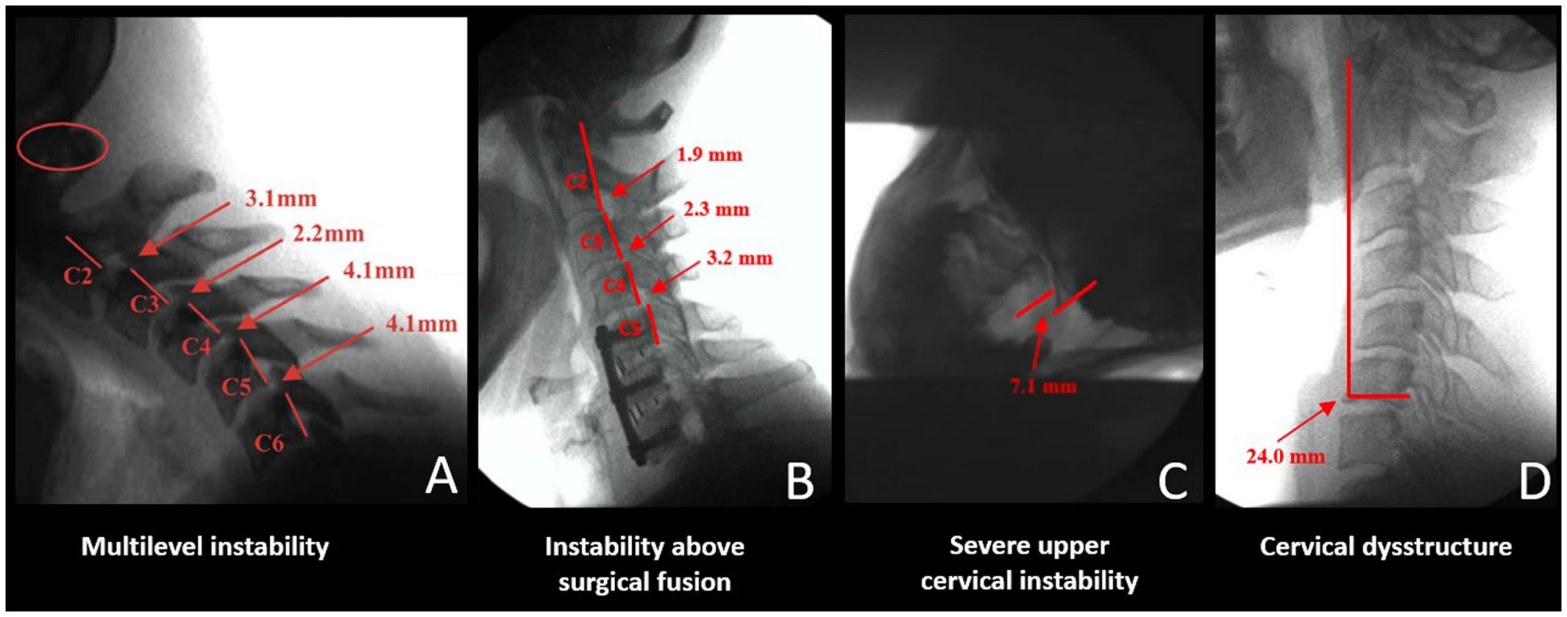
Common cervical digital motion (fluoroscopy) findings. **(A)** Multilevel cervical instability. **(B)** Instability above the level of a surgical or degenerative fusion. **(C)** Upper cervical instability at C1-C2 facet joints. **(D)** Cervical dysstructure.

DMX of the spine allows for a continuous and detailed examination of cervical spinal movement and allows unrestricted assessment of C0-C7 motion in multiple dimensions, including the sagittal, rotational, and frontal planes. DMX studies typically include actively moving the head and neck through protraction, retraction, flexion, extension, rotation, and lateral flexion. They show the functional integrity of the ligaments in the cervical spine, specifically the anterior and posterior longitudinal, supraspinous, interspinous, ligamentum flavum, transverse, alar, and facet capsular ligaments. The analysis of over 8,000 DMXs has found 4 common patterns^1^ (see Figure 7).

1. Extreme overall instability throughout the cervical spine from patients, typically females, with congenital hypermobility syndromes such as Ehlers-Danlos syndrome.
2. Instability above and below the level of prior surgical fusions or degenerative fusions, the latter being more prevalent in older males where the degeneration fusions (body fused or fixed the area by bridging osteophytes and disc degeneration) are in the lower segments, commonly C5-C7 and the unstable segments above it.
3. Severe upper cervical instability, especially at the C1-C2 level, can occur from a car accident, head trauma, birth trauma, or high-velocity rotational injury.
4. Cervical dysstructure: breakdown of the cervical curve with a very forward C1.

Severe anterior spondylolisthesis is generally recognized when horizontal translation is greater than 3.0–3.5 mm (56). While there are radiographic diagnostic criteria for lower cervical instability and extreme cases of upper cervical instability, especially those involving the transverse ligament where neurologic insult is current or imminent and surgery is warranted, the accepted diagnostic criteria for chronic upper cervical instability not requiring surgery are not so clear-cut (57–60). These classification systems are well-suited for medial atlanto-dens instability but do not evaluate in-depth lateral posterior LUCI involving the C0-C1 and C1-C2 facet joints and capsular ligaments. Many of these diagnostic criteria involve non-moving and non-weight-bearing imaging. Upright and motion scanning allows *changes to be seen* between positions and motions. Cervical ligamentous injury is documented when adjacent vertebral alignments are no longer maintained in certain positions or with certain motions.

## Neck vitals—ancillary diagnostic testing

Once the diagnosis of LCI, LUCI, or cervical dysstructure has been made by upright/motion radiography, ancillary diagnostic testing is performed to discover which pathophysiology is causing the most serious symptoms. We term the overall composition of pathophysiological tests that we perform the “neck vitals.” These tests include ocular and neck ultrasound, tonometry, pupillometry, and extracranial and transcranial Doppler ultrasound. They are used in conjunction with traditional testing methods such as brain, orbit, and cervical MRI, which typically have findings for conditions such as intracranial hypertension. Many of the MRI findings can be seen on ultrasound of the eye, including increased diameter of the optic nerve sheath, optic nerve sheath protrusion (length), retinal vein distention, and flattening of the posterior globe, which are correlated with intracranial pressure (ICP), intraocular pressure, and severity of papilledema (61–63). As MRI only shows the results of some cases of intracranial hypertension, the diagnosis can be missed (64). While obstruction of the various vascular fluid flows can be determined by MRI or CT angiogram, venogram, or CSF flow studies, as well as digital subtraction angiography, the main in-office methods we perform are transcranial and extracranial color duplex imaging. The net effect of CSF or venous flow obstruction can be intracranial hypertension (increased brain pressure), which can be found indirectly by various noninvasive testing methods, such as increased optic nerve sheath diameter. The neck vitals that pertain mainly to fluid flow analysis are discussed here.

## Optic nerve sheath diameter to document increased brain pressure

Normal pressure within the lumbar and brain CSF is noted to be 6–15 mmHg in the supine and lateral decubitus position, and slightly lower while upright, because of gravity (65). Of interest is that in neurological patients, ICP positively correlates with intraocular pressure, remembering that the eye is within the central nervous system (66).

Intracranial pressure monitoring using invasive methods (lumbar, brain, or cervical spine puncture) has been the gold standard for the evaluation of IBP, defined as a cranial pressure of greater than 20 mmHg, but limitations include its invasiveness and its potential complications, such as hemorrhage and infection (67). The optic nerves, as well as the other cranial nerves and spinal nerve roots, are surrounded by CSF in the subarachnoid space. As ICP rises, especially from CSF stasis, the CSF looks for a path of least resistance, one of which surrounds the optic nerve and can be evaluated by optic nerve sheath diameter measurements. Pressure on the optic nerve from CSF within the sheath and increased intraocular pressure can explain many of the visual disturbances, including loss of visual field, diplopia, and blurry vision symptoms with LCI and LUCI (24, 68).

The optic nerve emerges from the posterior part of the globe and appears as a hypoechoic linear structure with a hyperechoic border (nerve sheath). The outer rim should be included in optic nerve sheath measurements and should be measured 3.0 mm behind the posterior rim of the globe (where standard measurements are made.) Measurements in adults and children without IBP are typically around 3.0 mm+/−1.0 mm (69). While some studies in adults use a cutoff as low as 4.5 mm and as high as 6.0 mm as evidence for increased ICP, emergency rooms and most others use an optic nerve sheath diameter measurement of greater than 5.5–6.0 mm to diagnose intracranial hypertension or IBP (70–74).

**Figure 8:**
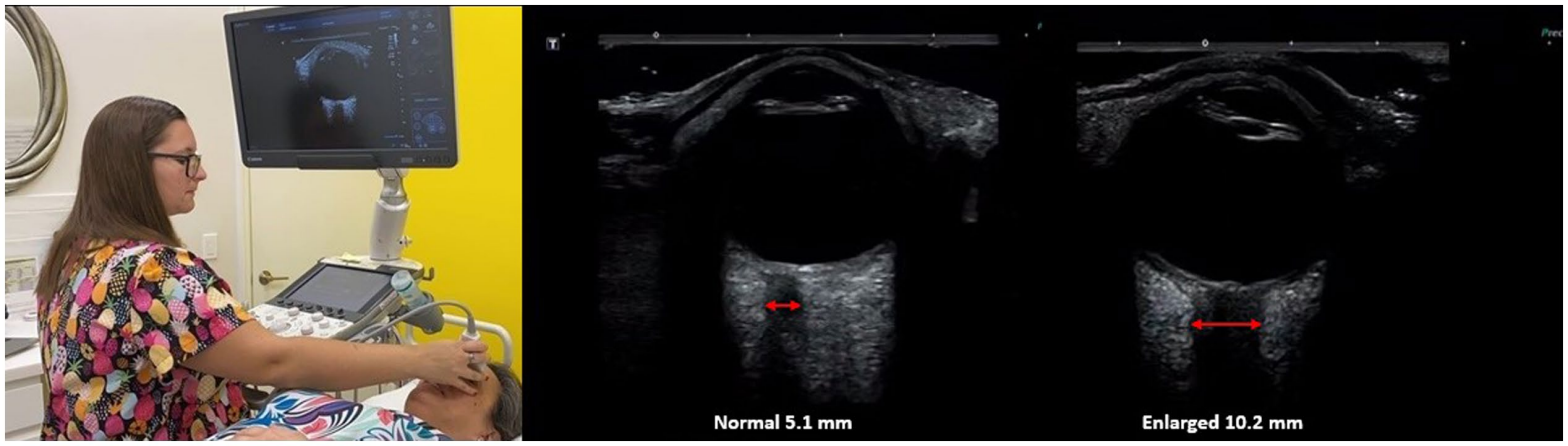
Ultrasound of eye to measure optic nerve sheath diameter, which is used as a marker for intracranial hypertension (increased brain pressure).

The optic nerve is well-suited for noninvasive evaluation of its diameter, as an ultrasound beam can travel unimpeded through the eyeball, which is mostly liquid. Historically, the optic nerve and its sheath diameter were noninvasively assessed by MRI and ophthalmoscopy, but transorbital ultrasound has recently emerged as a promising assessment tool (75). Ultrasound measurements of the optic nerve sheath diameter closely correlate with MRI measurements from *in vivo* and cadaver studies, and are reproducible (76, 77). Optic nerve sheath diameter has been shown to correlate with instantaneous assessments of ICP, including states of traumatic brain injury, and is even correlated with mortality (78–81). Typically, optic nerve sheath diameters over 6.0 mm are considered abnormal, and one of the evidences for IBP (see Figure 8).

## Comprehensive cervical ultrasound

Cervical ultrasound examination can reveal many findings that relate to cervical instability, including differences in size and flow velocities of carotid and vertebral arteries, and jugular veins, as well as evidence for dilated collateral vessels, lymphadenopathy, and measurements of pertinent nerve cross-sectional areas, including the vagus nerves (82–85). Vagus nerve degeneration (smaller cross-sectional areas) correlates with the amount of IJV compression. The differences between right and left sides are compared so the patient can be their own control and deviations from known normal values evaluated. Vascular obstructions are visualized in real time, including those in the carotid and vertebral arteries, and jugular veins, in various neck positions.

## Dynamic transcranial and extracranial Doppler ultrasound

**Figure 9:**
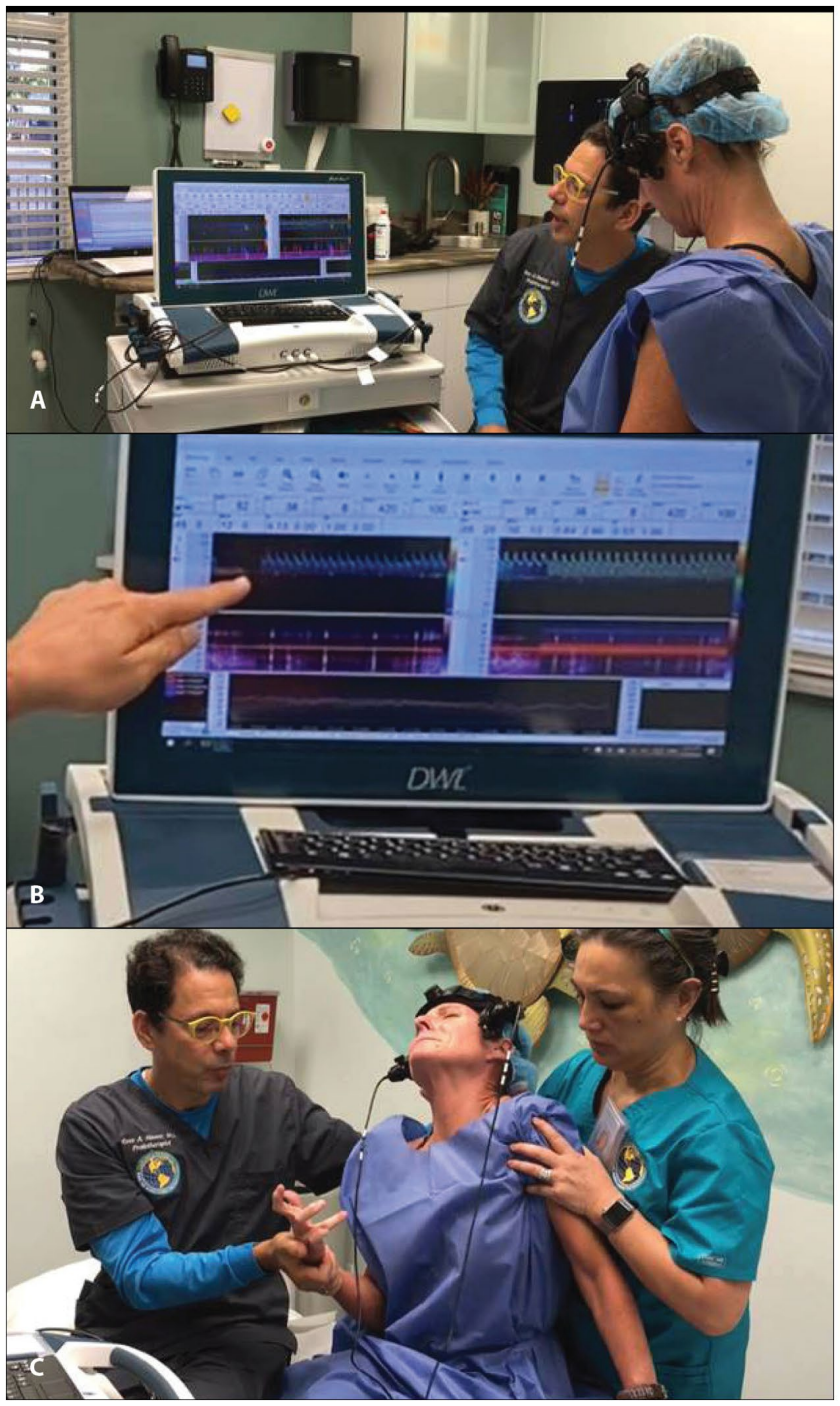
Motion transcranial Doppler monitoring. **(A)** Watching monitor while patient moves their neck. **(B)** Blood flow noted to be stopped in the middle cerebral artery. **(C)** Patient shown shortly after having a dystonic storm.

Transcranial Doppler (TCD) ultrasound provides real-time measurements of blood flow in the arteries that go to the brain, whereas extracranial Doppler (ECD) examines the veins and arteries in the neck as they go into and out of the brain. TCD has been called the “stethoscope for the brain” and ECD the “stethoscope for the neck.” These instruments can track moment-to-moment changes in blood flow to the brain from the vessels in the neck. They can assess the effect of interventions such as changes in neck position on brain blood flow (86–88) (see Figure 9). While the carotid and vertebral arteries can deform somewhat with neck motions such as rotation, there is not normally a significant change in carotid and/or vertebral artery blood flow with the upright position or neck rotation in patients with a cervical lordotic curve that is stable (89–92). TCD and ECD can be used to monitor the brain and neck blood flow for extended periods of time while holding a certain head/neck position. The blood flow in the brain, brainstem, neck, head, eye, and face can be compared in the supine, upright, and in various neck positions. A difference greater than 20% is considered significant to diagnose dynamic carotid artery stenosis or vertebrobasilar insufficiency, but changes in the waveform and pulsatility index (resistance to flow) can also be significant (93–96).

Blood flow constriction in the upper cervical spine is probable when LUCI is present due to the unique path the vertebral artery takes to go from the skull to the brain, where it is especially vulnerable to compression and stretch in the posterior neck at the level of the C1 and C2 vertebrae. The internal carotid artery is subject to compression and stretch, as it lies just anterior to the transverse processes of the C1, C2, and C3 vertebrae before entering the skull via the carotid canal.

While the real-time blood flow through the middle cerebral artery, the largest distal branch of the carotid artery, is measured by transcranial Doppler, so is pulsatility index, another noninvasive measurement for IBP (97, 98). Pulsatility index is a noninvasive method of assessing vascular resistance in the brain’s blood vessels, and it increases as intracranial pressure increases (99). Pulsatility index is defined as the difference between the peak systolic flow and minimum diastolic flow velocity, divided by the mean velocity recorded throughout the cardiac cycle, and a measurement greater than 1.20 typically means a brain pressure greater than 20 mmHg (100).

## Dynamic carotid and vertebral artery compression

The cervical spine structural anatomy affects brain health in many ways. LUCI can affect the 3 major fluid flows through the neck on their way to and from the brain: venous, arterial, and CSF. The more acute and serious of the fluid flows to be altered is arterial, as obstruction of blood flow to the brain will cause immediate ischemia and can be life-threatening. Venolymphatic and cerebrospinal fluid flow obstructions are generally intermittent, lead to IBP, and are more chronic and subtle. Sometimes there are both obstructions, as the carotid artery pulsations can encroach on the IJVs (101).

Dynamic carotid artery and vertebral compression are identified by significant changes in arterial velocities, compared to baseline readings (102). They can be documented by transcranial and extracranial Doppler examinations, and the exact place of compression can be identified in the process. The brain receives 20% of the cardiac output, with the internal carotid artery supplying the anterior 75–80% of the brain, and the vertebral arteries supplying the posterior 20–25%. Just 5 s of cerebral ischemia causes a reactive hyperemia via cerebral autoregulation, and reperfusion after 30 s of ischemia causes an increase in cerebral circulation by 3- to 4-fold (103). Cervical dysstructure has been shown to cause a decline in cerebral blood flow, which improved as lordosis was restored (104). Standard static tests, including CT and MR angiogram, and even extracranial ultrasound, can miss dynamic carotid artery compression unless a person’s neck is flexed and/or rotated (105, 106). Carotid artery compression symptoms can range from intermittent dizziness, vertigo, and lightheadedness to transient ischemic episodes and full strokes (107). This condition is diagnosed via dynamic transcranial Doppler and extracranial ultrasound evaluations, showing significant changes in the middle and anterior cerebral artery (end-branches of the internal carotid artery), and internal carotid artery blood flows with neck posture and upright position (105).

Arterial flow obstruction can be analyzed in a similar manner as disruption of nerve signals. In LUCI cases, the most commonplace obstruction of vertebral arterial blood flow is at and around the atlas, and the most common area of instability is the atlantoaxial joint (108). The vertebral artery most often gets compressed near the lateral mass of C1 on its way to penetrate the atlanto-occipital ligament and enter the skull through the foramen magnum.

While there are many causes of cervicogenic dizziness, perhaps the most underdiagnosed is vertebrobasilar insufficiency (109). Bow hunter’s syndrome is quoted in the medical literature as being rare, but it may just be rare because physicians do not suspect it can occur in little children as well (110, 111). Again, arteries, veins, nerves, and other structures around bones move with the bones. If the atlas is malrotated or unstable, adverse mechanical tension can occur on the vertebral artery. As the vertebral arteries supply the posterior brain, brainstem, and upper spinal cord, symptoms can be acute from posterior circulation ischemia and include vertigo, dizziness, drop attacks, dystonic storms, loss of vision, double vision, numbness, tingling, confusion, cognitive deficits, trouble swallowing, or nausea/vomiting. If the brainstem or posterior brain blood flow only becomes ischemic with rotational (or other) head motions, then CT angiogram or Doppler ultrasound examinations must be done in the symptomatic position.

Loss of cervical lordosis is associated with decreased vertebral artery values in lumen diameter, flow volume, and peak systolic velocity (112). Another study evaluated 256 patients with radiologically detected cervical instability, looking at its effects on vertebrobasilar blood flow. In 80% of the patients with instability of the cervical spine of >3.0 mm, there was cerebral circulation dysfunction (113, 114). A decrease in vertebral artery blood flow has been significantly correlated with a loss of cervical lordosis, while improvements in cerebral blood flow with cervical curve adjustments have also been documented to restore lordosis (104, 112).

## Internal jugular vein compression and increased brain pressure

The IJVs, also within the carotid sheath, exit the jugular foramen and run adjacent to the anterior border of the cervical vertebral bodies, especially the atlas, as fluid drains from the brain. The IJV is the main drainage port for the brain, especially in the supine position. Normal IJV cross-sectional area is 100 mm^2^ in the supine position and collapses to about 25 mm^2^ in the upright position because of negative pressure in the thoracic cavity with respiration (115–117). This IJV collapse increases cerebral pressure, helping maintain ICP in the upright position (118). The IJV cross-sectional area is easily measured under ultrasound.

Internal jugular venous compression is quite common and is one of the causes of chronic cerebrospinal venous insufficiency (119). In a study of 108 consecutive patients undergoing CT angiography for *presumed arterial obstruction*, 50% of them had IJV compression and 93% of the compressions were at the level of C1 (120). The IJV can also become compressed because of cervical instability, mandibular malposition, weary muscles, dysstructure, styloid bones, and even cervical collars (121, 122). Jugular venous outflow compromise is associated with many symptoms, as it can cause IBP, whose classic symptoms include head pressure, lightheadedness, headache, nausea, vomiting, visual loss, and sixth cranial nerve palsy, as well as swallowing difficulties, brain fog, photophobia, pulsatile tinnitus, and vertigo (123, 124).

ICP is normally kept in a very narrow range of 5–15 mmHg, with intracranial hypertension being a pressure greater than 20 mmHg. Intracranial hypertension is simply hypertension of the brain, or IBP. *It is important to understand that just as high blood pressure is the silent killer of the heart, high brain pressure is the silent killer of the brain.* Left untreated, IBP can have serious repercussions, including brain neuronal cell death and brain tissue atrophy (125, 126). The most likely cause of so-called idiopathic intracranial hypertension is venous hypertension from LCI, LCUI, and cervical dysstructure (127). Compression of the IJV is typically seen at the level of the atlas. Sometimes it is a combination of the atlas being too far forward from cervical dysstructure or moving too much because of LUCI, or because there are styloids also compressing the IJVs from the front. Symptoms of IJV compression from a styloid bone are worse with flexion and rotation to the side of the styloid.

At any one time, most of the volume of blood in the brain is in the venous system, trying to get out (128). While 32 quarts of fluids flow through the brain each day, 70–80% of them are contained in the brain’s venous system (128). In 94% of healthy people, the IJV is the main venous system through which blood exits the brain, with 6% of people draining less than one-third of the brain through the IJV (129). When the IJV is blocked by LUCI, especially in the upright position (for example, if C1 is unstable and sliding too far forward, compressing the IJV), the brain drains via vertebral veins, external jugular veins, as well as the deep cervical veins and lymphatics (130, 131).

Internal jugular venous compression can occur from something as simple as turning one’s head and wearing a cervical collar. Several animal and human studies have documented that by turning one’s head, the ipsilateral jugular vein can be significantly compressed (132–134). Besides causing compression of the IJV, head/neck rotation has been shown to block CSF flow and raise ICP (135, 136). The increase in ICP with neck motion was maximal with flexion and rotation, with ICP in 26% of the patients going over 50 mmHg when that position was held for 1–2 min (137).

Extracranial IJV compression is increasingly recognized as a cause of IBP, and occurs in the J3 segment at the level of the lateral mass (transverse process) of C1 and styloid process in up to 96% of cases (129, 138, 139). Styloid elongation greater than 30 mm is a risk factor for compression (140). This latter condition is known as Eagle syndrome, whose symptoms relate to compression of the carotid sheath contents (141, 142). Eagle syndrome can occur when extra tension is put on the stylomandibular and stylohyoid ligaments from FH-FDL and calcifies by pressure traction (143). The calcified styloid then compresses the carotid sheath, especially if it angles medially between it and the lateral mass of C1. It is easily seen by CT venogram with 3-D reconstruction of the cervical spine. This explains why lying down, mastication, laughing, and head retraction—all of which change the position of the mandible and C1 or a styloid bone, thus putting tension (stretching) on the carotid sheath—can worsen symptoms due to carotid sheath compression (144).

**Figure 10:**
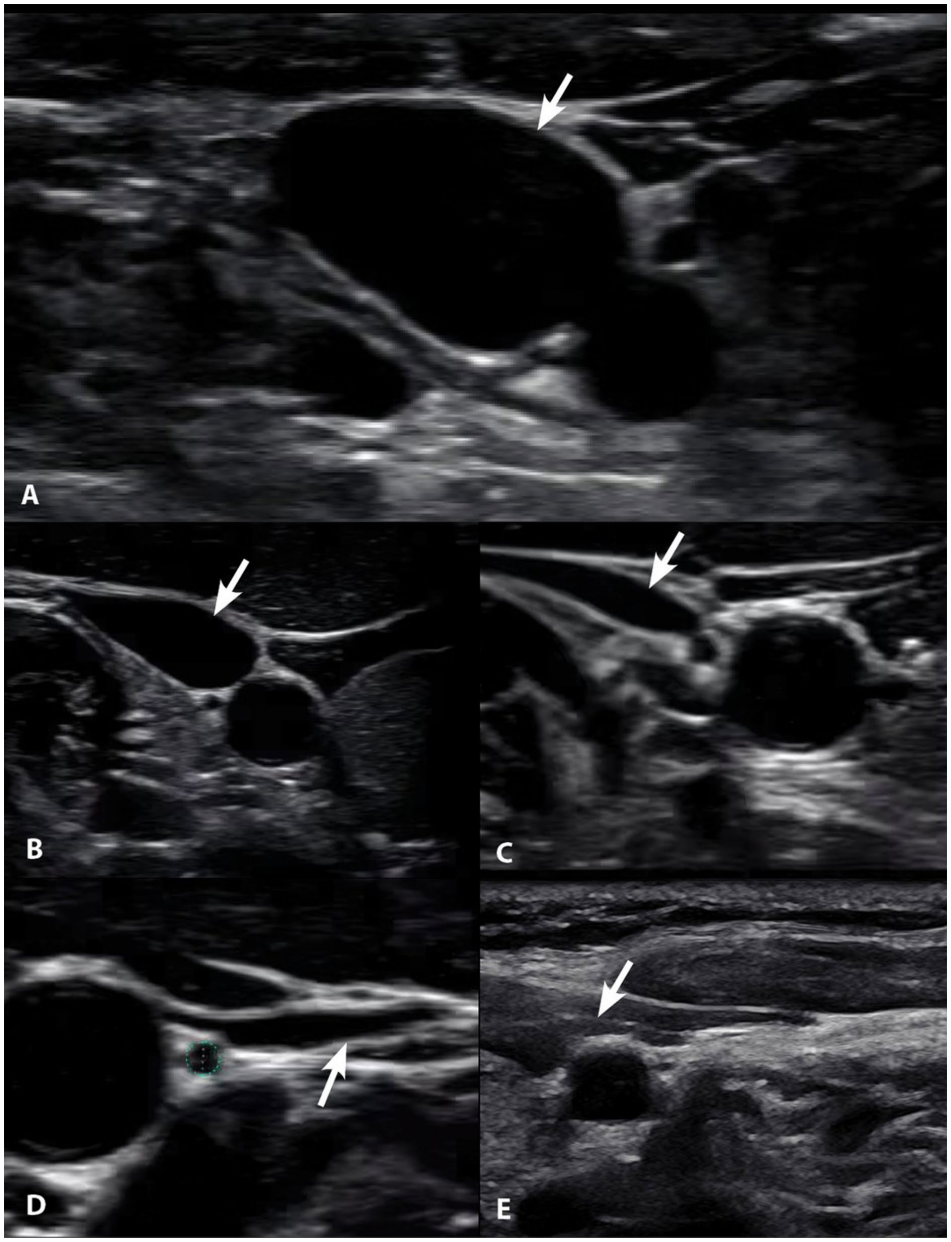
Degrees of internal jugular vein compression as seen on ultrasound examination of the neck. **(A)** Normal, “open” internal jugular vein. **(B)** Slightly compressed. **(C)** Moderately compressed. **(D)** Severely compressed. **(E)** Completely closed. It is internal jugular vein compression (arrows), especially with upright posture and neck motions, that leads to intracranial hypertension (increased brain pressure).

Compression of the carotid sheath, which contains the IJV, can be caused by a narrowing of the space between the lateral mass of C1 and the styloid (3.7 mm of narrowing, in one study) known as the atlantostyloid interval (145). The diameter of the IJV correlating with reduced size of the atlantostyloid interval and the asymmetric larger area of the lateral mass of the atlas on the side of compression (by 40%) suggests LUCI as a reason for the bony size discrepancy (146). Since the styloid is anterior to the carotid sheath, retraction of the head should increase symptoms if an enlarged styloid is the cause of IJV compression; protraction of the head/jaw therefore relieves symptoms, as the atlantostyloid interval increases with that motion. While the gold standard to diagnose IJV compression has been CT venograms, in many cases it is possible for CT or MR venograms done in the supine neck neutral position to miss the IJV compression, as the atlantostyloid interval is narrowed maximally by contralateral head rotation (132). Static neutral scans do not consider compression of the carotid sheath with rotation, flexion, or extension of the neck. Ultrasound evaluation while moving the head in different positions can show the narrowing of the IJV at the site of compression (see Figure 10).

A proposed cervical ligament etiology of IBP is the 3:1 to 18:1 female preponderance, often relieved by lying down, and the fact that neck flexion and rotation can increase ICP by almost 9 mmHg (147–149). In fact, 90% of the patients are females of childbearing age (the most flexible adults), again possibly pointing to cervical instability as a cause (150).

## Treatment principles

Successful treatment is determined in large part by correctly identifying the underlying cause of symptoms and diseases. When the etiology is presumed to be structural in nature, treatment outcomes directed at the dysfunctional structure (dysstructure) will likely be more successful than chemical ones (medications). In regard to treatment of ICP, the main target of lowering the pressure in IBP is currently reached by medications such as acetazolamide, which acts as an inhibitor of the sulfonamide-sensitive carbonic anhydrases and reduces CSF secretion by approximately 50% (151). Weight loss combined with acetazolamide can be used, as well as ventricular shunting when ICP becomes severely elevated with significant symptoms (152, 153). The prognosis with these approaches is limited, as they do not resolve the condition (154).

As the most likely cause of ICP as presented here is structural, treatments should be directed at improving the structure. Brain fluid outflow is compromised by venous obstruction from both the immediate and long-term effects of LCI, which include vertebral malalignment, curve breakdown, and instability. A *structural* treatment program would therefore be necessary. Components of this program could include dynamic orthoneurological (chiropractic) adjustments for vertebral subluxations, curve correction by chest and head weights and therapeutic exercises, and Prolotherapy to tighten injured incompetent ligaments. It should be noted that while vertebral subluxations or misalignments—structural asymmetries in vertebral anatomy causing clinical symptoms—can be found objectively on radiographic or physical exam assessments, their clinical significance, though recognized by the World Health Organization, is not universally accepted (155, 156). When clinically significant, the correction is often performed by improving the biomechanics of the cervical curve and its structure, so the fluid flow and nerve signal transmission to and from the brain are improved, along with proprioception. This is confirmed by a lowering of the optic nerve sheath diameter and pulsatile indexes, documentation improvement of ICP, and thus brain health parameters.

**Figure 11:**
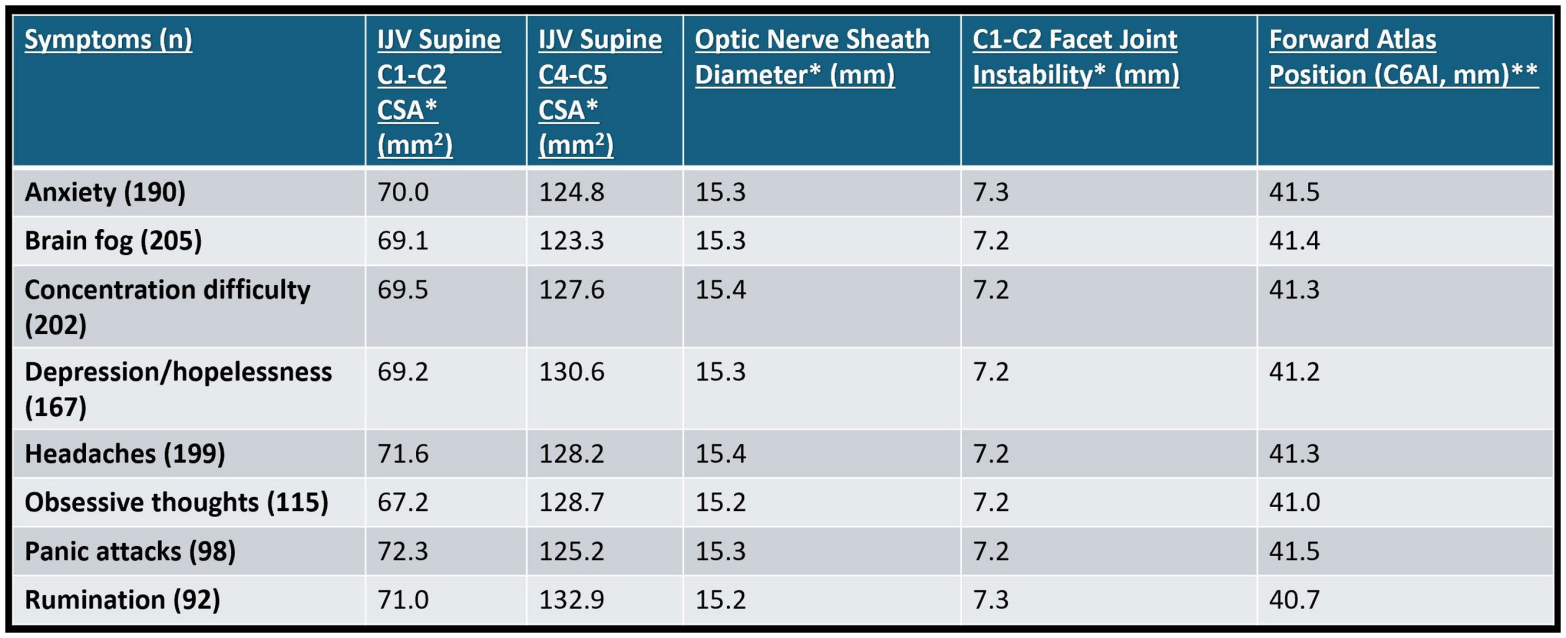
Summary of cervical structural analysis and ultrasound measurements of internal jugular veins (IJVs) and optic nerve sheath diameters (ONSDs) in 227 consecutive patients aged 20–50 who came to an outpatient neck center and had at least 1 of the 8 symptoms above, with no history of trauma or etiology of symptoms. * Mean bilateral total internal jugular vein cross-sectional areas (CSAs), ONSDs, and C1-C2 facet joint instability in patients with various brain/mental health symptoms. Normal for IV CSA >180 mm^2^, ONSD is <12.2 mm, and optimal C1 overhang on C2 in lateral flexion, open mouth is <2–4 mm. ** C6A1 = C6-atlas interval, horizontal distance in the sagittal plane of the posterior inferior C6 vertebra to anterior atlas (optimal is <10 mm). Measures how far forward the atlas (C1) is in relation to lower cervical vertebra (C6).

The cervical structural, IJV, and optic nerve sheath diameter measurements for 227 consecutive patients aged 20–50 from January 1, 2022 to June 30, 2022, with no obvious cause for at least 1 of 8 specific brain or mental health symptoms—anxiety, brain fog, concentration difficulty, depression/hopelessness, headaches, obsessive thoughts, panic attacks, and rumination on traumatic events—were taken (see Figure 11). Some 96.2% of patients had an abnormal optic nerve sheath diameter (>6.1 mm). The cervical instability and dysstructure found are presumed to “simply” be from FH-FDL with computer and cell phone usage.

## Dynamic structural medicine

Body mechanics in health and disease are an integral part of medical specialties such as orthopedic surgery, physical medicine and rehabilitation, neurology, and osteopathic and chiropractic medicine. It involves how the human frame moves in 3-D space and its effect on joint and neurological structures, as well as organ systems and their functions. A more modern term to describe this field is “dynamic structural medicine,” defined as how human structure influences health and disease. “Dynamic” embodies the constant state of change that occurs in the human body to maintain homeostasis in various postures, positions, and motions. A coordinated and balanced effort between the musculoskeletal, neurologic, and vascular systems is required to maintain a healthy upright posture. It is underappreciated how subtle changes in spinal curvature, posture, and stability affect this delicate balance.

Without normal spinal alignment and movement, neurologic structures that traverse through the neck are at risk. Once alignment, curve, or stability are compromised, the body starts making compensatory changes down the spinal kinetic chain, with changes typically progressing from the neck down (157). Compared to people with normal lordotic curves, those with kyphosis note greater incidence of neck pain and scoliosis, disc herniations that are more severe, increased incidence of spinal stenosis with and without myelopathy, and spinal cord tensions associated with elevated intramedullary pressures (158–161). Missed dynamic cervical spine instability is associated with spinal cord compression, most commonly at the C5-C6 segment and cervical segments with greater disc bulge, more severe disc degeneration, greater angular motion, segmental kyphosis, and developmental stenosis (162).

Static and dynamic DMXs help determine which of the 3 variables (alignment, curve, and/or stability) are involved in a patient’s pathology, but typically one needs motion to show instability. By taking static and motion x-rays in the various neck planes, a picture of the 3-D cervical anatomy is revealed. The primary structural lesion is identified as causing the patient’s symptoms, and then the correct therapeutic remedy is applied. In cases of forward head posture or poor cervical curve in the absence of instability, restoration of the curve is crucial for overall health.

## Neck reconstruction therapy

**Figure 12:**
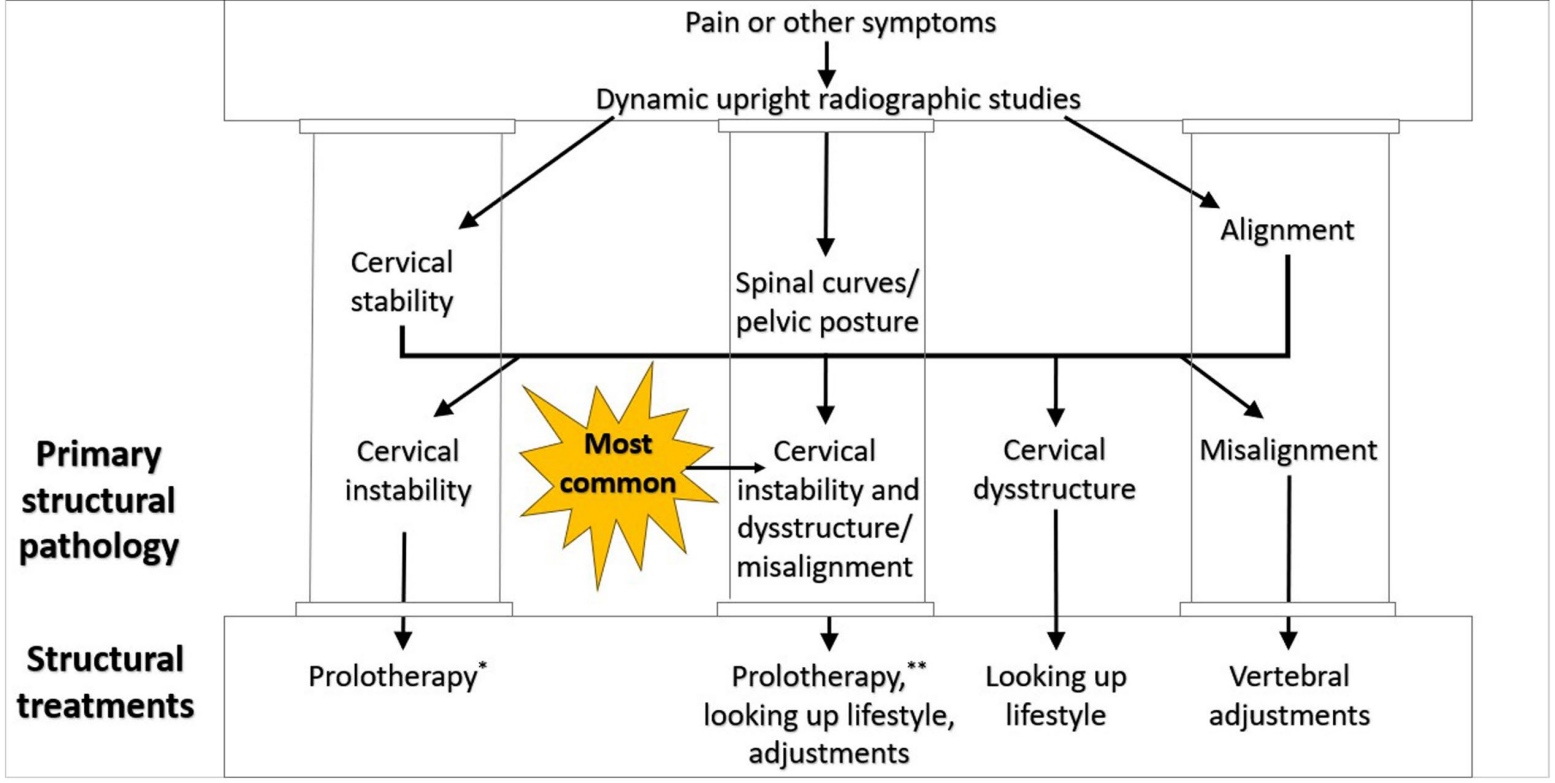
Cervical treatment recommendations based on dynamic upright radiographic studies. Patients often have a combination of cervical instability, cervical dysstructure (breakdown of cervical curve), and misalignments, the 3 pillars of cervical structural health. * Some extreme cases of instability require surgery or other methods. ** Optimizing cervical curve is multifaceted and can include ergonomics, physical therapy and exercise, and many other physical medicine techniques.

Ideal structural integrity of the cervical spine involves 3 primary parameters: alignment, curve, and stability. LCI can result in misalignment (subluxation), curve deformation, and ligamentous instability. In many ways, they are a continuum of the progressive nature of LCI, so treatment is directed at each part of the triad (see Figure 12).

## Dynamic orthoneurological correction (upper cervical adjustment)

Specific spinal manipulations (or adjustments) are the basis for much of the chiropractic profession (163). Traditional subluxations are diagnosed via static x-rays and physical examination, while dynamic orthoneurological corrections put heavy emphasis on dynamic 3-D anatomy, including spinal curves, utilizing motion x-rays, and taking into account the diagnostic neck vital tests to determine the primary lesion. Corrections are of little force, especially for the upper cervical region, with an activator or adjustment tool that an upper cervical chiropractic specialist would use, and not with high-velocity rotational forces.

Misalignments, especially of the atlas and axis, can have significant effects on compressing the carotid sheath. They are easily diagnosed on DMX and can compress the carotid sheath on the side of the rotation (with specific head positions and motions), causing characteristic pathophysiology and symptoms. The correction is often performed from a mostly anterior to posterior approach, as the cervical deformation with LCI is anterior.

## Cervical curve correction

While a study published in 1960 demonstrated that only 3 out of 180 patients (<1.7%) had cervical kyphosis, comparing it to recent studies shows that 30–40% of the general population has cervical kyphosis (21, 164). As those who have kyphosis are more prevalent in younger groups than those with straight or lordotic necks, and the fulcrum of the kyphosis is at C4-C5—the same fulcrum as neck flexion—clearly, the FH-FDL is involved (165, 166). One can then infer some connection between the alarmingly rising rates of cervical kyphosis and the increased use of cell phones and computers, especially in younger populations.

When cervical sagittal anatomy is optimized, symptoms including dizziness and neck pain resolve (167). Cervical curve correction may be initiated for many reasons, including structural, physiological, and symptomatologic (168). Indications that a patient may require cervical curve correction therapy are if observation and/or imaging show a significant forward head posture, or if the patient exhibits significant reversal of their cervical lordosis (169, 170). Other less obvious indicators that curve correction will be required for a successful treatment regimen are things like pooling of CSF at a certain level in the cervical spine with associated structural origin (as visualized on MRI) or flattening of the IJV in front of the upper cervical vertebrae.

**Figure 13:**
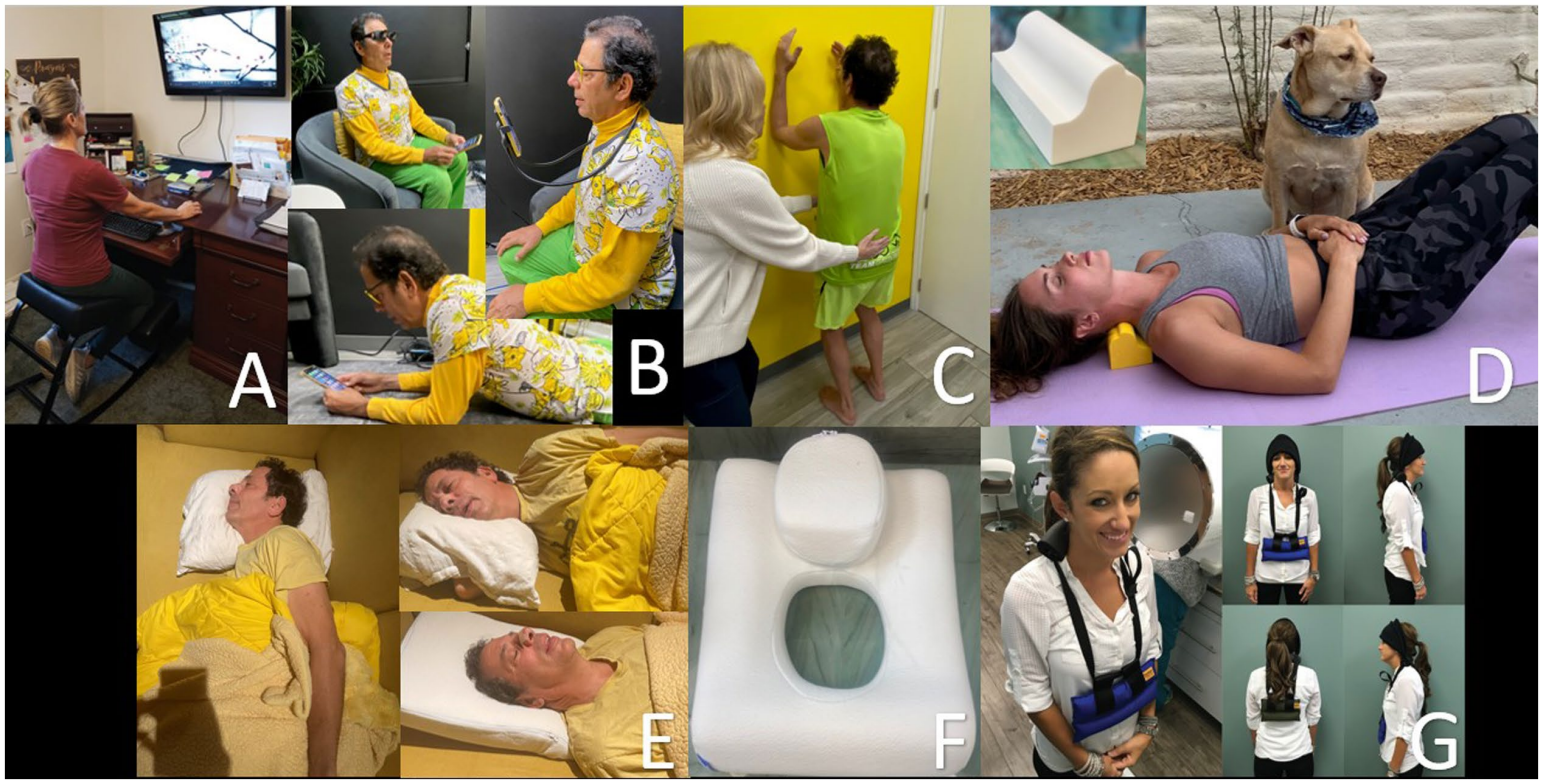
Methods to improve one’s cervical curve. **(A)** Proper workstation. **(B)** Proper looking-down posture. **(C)** Postural exercises. * **(D)** Laying on Denneroll^®^. **(E)** Proper sleep position. **(F)** Pillow with cut-out. **(G)** Wearing neck weights. * Postural restoration institute.

Cervical curve correction can be as simple as changing one’s computer setup to be looking up, doing various postural exercises, or changing their sleeping position to one that opens up the jugular veins maximally (171). In our opinion, lying on a cervical orthotic such as the Denneroll^®^ or using patient-specific weights in a standing or weight-bearing position can also help (see Figure 13). The patient can be x-rayed periodically to make sure the cervical curve is correcting, and the opening of the jugular veins can be verified with ultrasound.

## Prolotherapy

Prolotherapy is often referred to as a regenerative injection technique that is used to stimulate repair of injured soft tissues, including ligaments. Our definition of “Prolotherapy” is: proliferative injections onto the bone at ligament attachments that strengthen and tighten ligaments to resolve joint instability. It is our opinion that the progress of Prolotherapy can be shown by a decrease or resolution of the instability on repeat DMXs; what can also be shown is an improvement in the neck vitals.^2^

Ligamentous tissue is often slow to heal on its own due to a lack of blood supply, which explains why people can develop chronic cervical instability that worsens over time. Prolotherapy to the cervical spine involves injecting an irritating solution (often dextrose) at ligament and tendon attachment sites that creates a mild inflammatory response, initiating the body’s natural healing cascade targeted at poorly vascularized tissue (172, 173). Load-bearing capacity, mass, tensile strength, and elasticity of collagenous connective tissues are increased. This initiates responses such as fibroblast and platelet activation, which repair and reinforce injured connective tissue.

**Figure 14:**
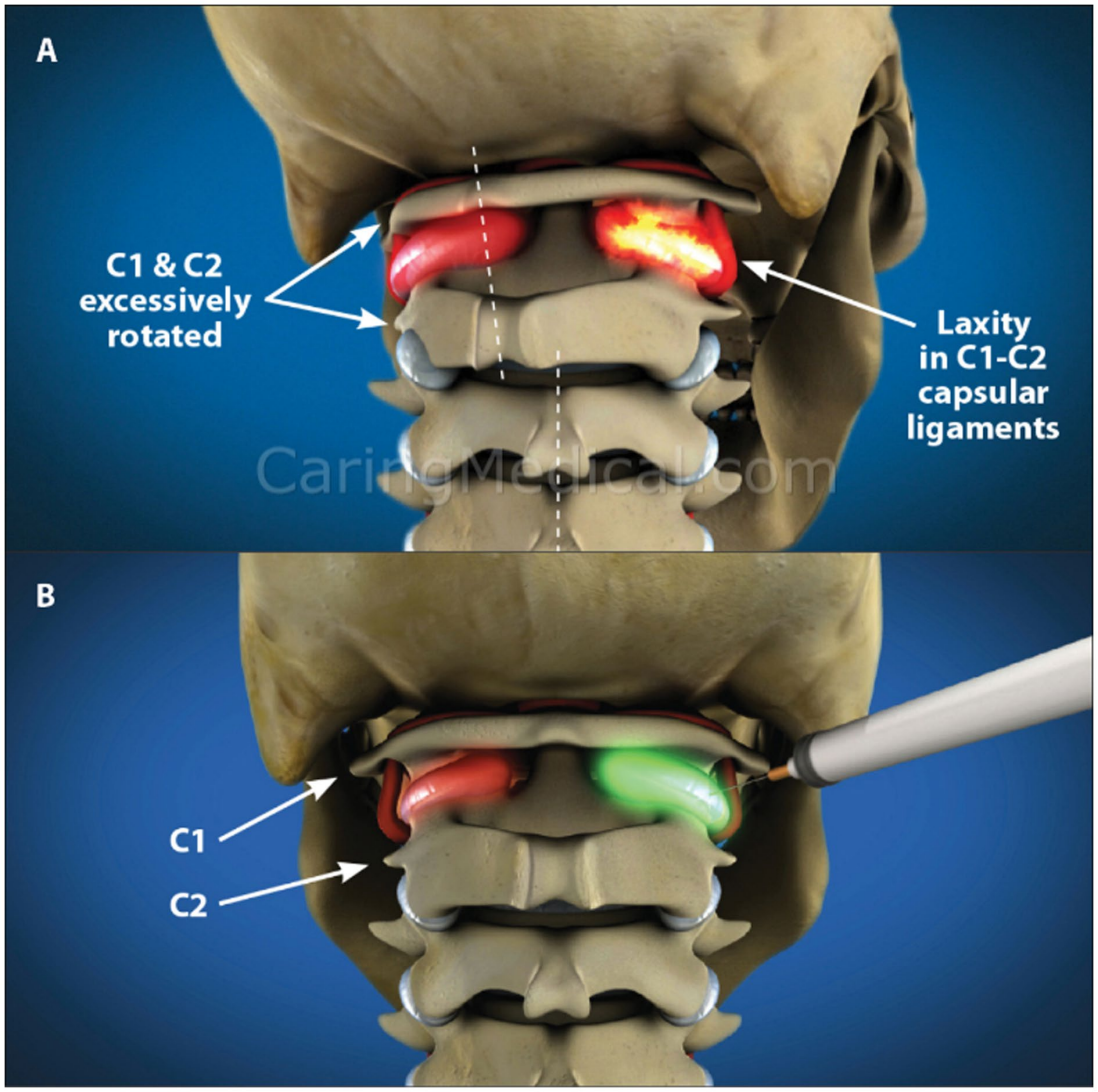
3-D rendition showing upper cervical instability with motion and treatment by prolotherapy. **(A)** Upper cervical instability. **(B)** Prolotherapy to the C1-C2 facet joints.

Prolotherapy was developed by George Hackett, MD in the 1930s with the primary target to treat potential pain sources within connective tissue by either proliferation of new cells or the improvement in the health of existing cells (174). The most studied type of Prolotherapy utilizes dextrose, but other solutions may be used, such as platelet rich plasma, adipose tissue, bone marrow aspirate, or other minerals or fatty acids. Evidence that Prolotherapy induces the repair of ligaments and other soft tissue structures, including tendons, has been reported in both animal and human studies (175–177). Prolotherapy injections in the neck are directed at the posterior ligament complex, especially the capsular ligaments surrounding the facet joints (see Figure 14).

In independent studies, Hackett and others reported significant successful outcomes in using Prolotherapy to treat ligament injuries, especially in those with cervical ligament injury-related symptoms, such as headache or whiplash-associated disorder (178–180). Additional clinicians documented significant symptom relief with cervical Prolotherapy directed at the posterior ligamentous complex, including the capsular ligaments (14, 181–183). Prolotherapy, in the absence of dynamic orthoneurological correction and cervical curve correction, is promising for those suffering from LCI. When coupled with loss of the lordotic curve and cervical malrotations, LCI could be reversed using proper structural treatment aimed at resolving targeted structural defects.

## Case study

**Figure 15:**
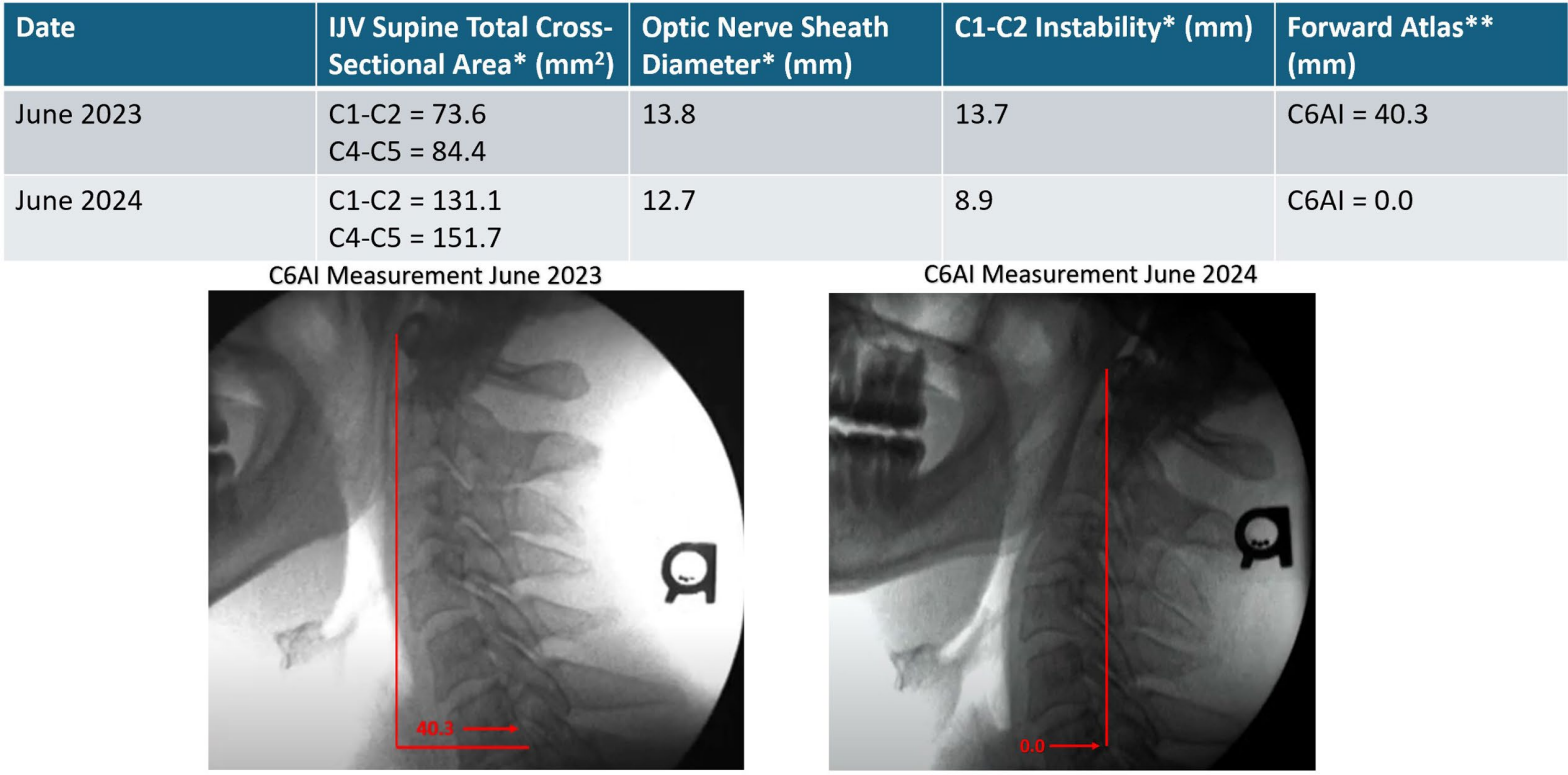
Improvement in patient’s cervical curve structure correlates with opening of his internal jugular veins and reduction in optic verve sheath diameter. Over the course of a year, his brain and mental health symptoms improved significantly. * Mean bilateral total internal jugular vein (IJV) cross-sectional areas (CSAs), optic nerve sheath diameters (ONSDs), and C1-C2 facet joint instability in patients with various brain/mental health symptoms. Normal for IJV CSA is 180 mm^2^, ONSD is <12.2 mm, and optimal C1 overhang on C2 in lateral flexion, open mouth is <2–4 mm. ** C6A1 = C6-atlas interval, horizontal distance in the sagittal plane of the posterior inferior C6 vertebra to anterior atlas (optimal is <10 mm). This measurement shows how far forward the atlas (C1) is in relation to the lower cervical vertebra (C6).

A 51-year-old man with a long history of anxiety, depression, dissociative disorder (depersonalization/derealization), panic attacks, brain fog, memory decline, concentration difficulty, chronic fatigue, low stress tolerance, chronic neck pain, and head pressure has worked as an engineer for 30 years, putting in long hours hunched in front of a computer screen. He has seen many healthcare providers, including psychiatrists, psychologists, and chiropractors, but his symptoms continue to progress. He admits to self-manipulating his neck many times a day. Initial cervical structural testing revealed severe cervical dysstructure and upper cervical instability, causing compression of the carotid sheath. He was told to stop self-manipulating his neck and was educated on improved ergonomics for his workstation, so he bought a rocker-bottom chair and standing desk and raised his computer height significantly. His best positions to sleep in were on his left side with his head extended or with a cut-out (Denneroll^®^) pillow, as they opened his IJVs compared to his normal chin-down sleeping position. He was initially told to lay on a Denneroll^®^ for 5 min every hour (with improvements in curve, this time went down to 20 min per day), started exercises to strengthen his neck musculature, and was given Prolotherapy for his LCI. Over the course of 1 year, during which he received 6 Prolotherapy sessions, we noted a significant increase in his IJV cross-sectional areas and improvement in his neck curve (see Figure 15). Upon his last visit to the clinic in June 2024, he noted no more dissociative episodes nor panic attacks, and approximately 80–90% subjective improvement in his brain and mental health symptoms. He continues his cervical curve correction program.

## Summary

The FH-FDL from excessive time spent looking at computers, laptops, and cell phones causes the slow stretching of posterior ligaments in the cervical spine, especially the capsular ligaments. As cervical ligaments stretch out, they can no longer handle the forces of the head on the neck and the normal cervical curve starts to break down, a process called cervical dysstructure. Clinical ligamentous cervical instability results, which is an inability of the cervical ligaments to maintain individual, adjacent, or global vertebral alignment when subjected to increased forces by various postures, positions, and/or motions that alter bony, soft tissue, and/or neurovascular alignment and function such that symptoms result. The cervical lordotic curve becomes straight and then kyphotic as head forces go in front of the cervical vertebrae, causing the condition to be progressive. This change in cervical curve can affect everything that traverses the neck, including vital neurovascular structures. Cervical muscle spasms, facet joint swelling, and degeneration occur to try and limit destructive neck motions. When the body runs out of compensatory mechanisms to limit forces and destruction, neurologic and vascular compromise and injury occur, causing symptoms.

Dynamic structural medicine explains how human posture and motion affect body and brain health and disease. Detrimental changes in the neck curve—cervical dysstructure from ligamentous cervical instability—cause compression of the carotid sheath, narrowing the jugular vein and stretching and putting pressure on the vagus nerve, potentially causing brain and body disease, respectively. The most serious symptoms occur when brain health is affected by the IJVs and the carotid and vertebral arteries becoming compressed, causing intracranial hypertension and arterial insufficiency or ischemia, respectively. These conditions can cause myriad symptoms from neck tension, headache, blurry vision, dizziness, tinnitus, and ringing in the ear, to personality changes and many others. Because the neurovascular compression often occurs in the upright position or with specific neck motions, traditional MRI or CT scans in the supine position can miss it. Cervical structural analysis is performed in the upright position in different head and neck positions with DMX and cone beam CT. A neck vitals analysis is done to document the pathophysiology caused by the LCI.

As presented here, the most likely cause of IBP is structural. This reaffirms that a *structural* treatment program should be prescribed that is aimed at correcting incompetent structures of the cervical spine. Ligamentous cervical instability is often the missing structural cause for many chronic conditions associated with the cervical spine and neck’s relevant anatomy. Instability, along with loss of the cervical lordotic curve, can be the cause of increased brain pressure and many associated chronic conditions. Treatments directed at cervical vertebral malalignments (subluxations), curve dysstructure (breakdown), and instability include dynamic orthoneurological (chiropractic) low-force adjustments, curve correction and therapeutic exercises, and Prolotherapy, respectively.

## Data Availability

The original contributions presented in the study are included in the article/supplementary material, further inquiries can be directed to the corresponding author.

## Footnotes

^1^Analysis of patients coming to our outpatient Hauser Neck Center. As of November 1, 2024, final statistical analysis is being completed for scientific publication.

^2^Opinion based on 8,000 DMXs and data that are forthcoming in subsequent articles after this overview article.
